# Endothelin inhibits renin release from juxtaglomerular cells via endothelin receptors A and B via a transient receptor potential canonical‐mediated pathway

**DOI:** 10.14814/phy2.12240

**Published:** 2014-12-18

**Authors:** M. Cecilia Ortiz‐Capisano

**Affiliations:** 1 Hypertension and Vascular Research Division, Department of Internal Medicine, Henry Ford Hospital, Detroit, Michigan

**Keywords:** Calcium, endothelin, ETA, ETB, renin, transient receptor potential canonical

## Abstract

Renin is the rate‐limiting step in the production of angiotensin II: a critical element in the regulation of blood pressure and in the pathogenesis of hypertension. Renin release from the juxtaglomerular (JG) cell is stimulated by the second messenger cAMP and inhibited by increases in calcium (Ca). Endothelins (ETs) inhibit renin release in a Ca‐dependent manner. JG cells contain multiple isoforms of canonical transient receptor potential (TRPC) Ca‐permeable channels. The proposed hypothesis is that endothelin inhibits renin release by activating TRPC store‐operated Ca channels. RT‐PCR and immunofluorescence revealed expression of both ETA and ETB receptors in mouse JG cells. Incubation of primary cultures of JG cells with ET‐1 (10 nmol/L) decreased renin release by 28%. Addition of either an ETA or an ETB receptor blocker completely prevented the ET inhibition of renin release. Incubation with the TRPC blocker (SKF 96365, 50 *μ*mol/L) completely reversed the Ca‐mediated inhibition of renin release by ETs. These results suggest that endothelin inhibits renin release from JG cells *via* both ETA and ETB receptors, which leads to the activation of TRPC store‐operated Ca channels.

## Abstract (precis)

This manuscript reexamines the relationship between endothelin and renin secretion from the juxtaglomerular cells, demonstrates the presence of endothelin A and B receptors on the cells. Furthermore, it describes a novel TRPC channel linking activation of the ETA/ETB to the calcium‐mediated inhibition of renin.

## Introduction

Renin is the rate‐limiting step in the production of angiotensin II: a critical element in the regulation of blood pressure and in the pathogenesis of hypertension. Renin is produced by, stored in and released by juxtaglomerular (JG) cells (Barajas 1979; Hinder et al. 1999) in the renal cortex. Two main intracellular second messenger systems are known to regulate renin secretion: stimulation of renin release by the cyclic nucleotide, cyclic adenosine monophosphate (cAMP), and inhibition of renin secretion by increased intracellular calcium (Ca; Churchill and Churchill 1982; Antonipillai and Horton 1985; Fray and Park 1986; Harding et al. 1997).

The endothelin (ET) system comprises a family of three isopeptides (ET‐1, ET‐2, and ET‐3). ET‐1 is a major renal peptide that binds to ETA and ETB receptors (Guan and Inscho 2011). In vitro, ETs inhibits renin release in a Ca‐dependent fashion (Ackermann et al. 1995; Ritthaler et al. 1995). It has been reported that ETs act on JG cell ETB receptors, activating phospholipase C (PLC) and inositol triphosphate (IP3) in JG cells, resulting in Ca release from intracellular stores and subsequent Ca entry (Ritthaler et al. 1995); a pathway known as store‐operated Ca entry (Parekh and Putney 2005). However, any evidence of ETA or ETB expression of in JG cells is indirect as there are no data documented anatomical expression of these receptors in JG cells, and little information regarding an actual mechanism by which Ca mediates ET inhibition of renin release.

The transient receptor potential canonical (TRPC) channel proteins have been identified as downstream molecules in a G‐protein‐coupled receptor signaling pathway, and are involved in a variety of cell functions due to their ability to regulate intracellular calcium signaling (Nilius and Owsianik 2011). Among those, TRPC1, 4 and 5 isoforms are known to function as store‐operated Ca channels. Endothelin (ET) 1, *via* ETB receptors, could activate the phospholipase C (PLC)/IP3 signaling pathway, potentially stimulating store‐operated Ca channels (Salido et al. 2009). Recently published data from this laboratory described TRPC 3 and 6 expression in isolated JG cells and their involvement in Ca‐mediated inhibition of renin release in an adenosine‐dependent system (Ortiz‐Capisano et al. 2013a). TRPC1, TRPC4, and TRPC5, proposed to be possible candidate channels for SOCE (Liao et al. 2008), might be expressed in JG cells as well. Because of these previous observations, I hypothesized that endothelin, *via* ETB receptor activation, inhibits renin release by triggering the TRPC store‐operated Ca channels (isoforms 1, 4, and/or 5) to increase intracellular Ca.

## Materials and Methods

### JG cell preparation

This study conforms to the *Guide for the Care and Use of Laboratory Animals* published by the National Institutes of Health, the protocol was approved by the Institutional Animal Care and Use Committee of the Henry Ford Health System.

#### Isolation of mouse JG cells

In all of the following protocols, primary culture of mouse isolated juxtaglomerular (JG) cells were used, based on the methods of della Bruna et al. (Kurtz et al. 1988; della Bruna et al. 1991) which have been extensively modified to improve the harvest, purity, and stability of the primary culture, as previously described in detail (Ortiz‐Capisano et al. 2007b,c; Mendez et al. 2011). JG cells obtained from C57BL/6NJ male mice were incubated at 37°C in a humidified atmosphere containing 5% CO_2_ in air. After 48 h of incubation, the culture medium was removed, and replaced with 250 *μ*L of fresh prewarmed serum‐free culture medium containing 1.2 mmol/L calcium, with the phosphodiesterase inhibitor, 3‐isobutyl‐1‐methyl‐xanthine (IBMX; Sigma, St Louis, MO); 0.1 mmol/L dissolved in Dimethyl sulfoxide (DMSO; Sigma, St Louis, MO), plus the various drugs to be tested, as described below. JG cells were incubated for 1 h, after which the supernatant was collected, centrifuged to remove any cellular debris, and assayed for the activity of renin released into the medium (see below). Every experimental day, JG cells from four donor mice were pooled to produce four wells of a primary culture, and controls and treatment run in parallel. Each day, experiments represent an *n*‐value of 1.

### JG cell ETA and ETB receptor expression

#### RT‐PCR for ETA and ETB receptors in JG cells

RT‐PCR for the ET receptors was run using a preparation of pipette‐selected isolated mouse JG cells to minimize non‐JG cells contamination. Using a preparation previously described (Ortiz‐Capisano et al. 2007b,c), individual JG cells were picked up using a 1 mL pipette under a Fisher Scientific inverted microscope at a 40× magnification. A suspension of mouse liver cells was used as a positive control (Lee and Ahn 2008). A “no‐template” control was used as a negative control. Isolated JG cells were resuspended in 1 mL of Tri reagent. Likewise, mouse liver was homogenized in 1 mL Tri reagent. One microgram of extracted total RNA was reverse transcribed at 37°C using Omniscript (Qiagen, Valencia, CA) and 2 *μ*L of mixture was taken for subsequent PCR. The following primers, (NCBI accession number: NM_010332.2) were used to detect the ETA receptor (Lee and Ahn 2008): Sense 5′ acggtcttgaacctctgtgc3′ and Antisense 5′ agccaccagtccttcacatc 3′. To detect the ETB receptor, the following primers, (NCBI accession number: NM_007904.4) were used (Lee and Ahn 2008): Sense 5′ ggtatgcagattgctttgaatgat 3′ and Antisense 5′ tgtggattgctctggtcataca 3′. PCR was performed under the following conditions: 94°C for 2 min; 40 cycles of 95°C for 30 sec, 60°C for ETA receptor and 58°C for ETB receptor for 40 sec, 72°C for 1 min, followed by a final extension at 72°C for 5 min. The reaction products were then held at 4°C. PCR products were run on a 2% agarose gel in 1× TBE. A2aR and A2bR give a PCR product at 260 and 231 bp, respectively (Lee and Ahn 2008).

#### Immunolabeling of ET receptors A/B and renin in JG cells

We placed primary cultures of JG cells on Poly‐D‐Lysine‐coated coverslips for 48 h. The medium was then removed and the cells fixed for 30 min with freshly prepared 4% paraformaldehyde diluted in phosphate buffer solution (PBS), then washed with tris‐buffered saline tween (TBST) three times for 5 min each. The fixed cells were permeabilized with 0.2% Triton X‐100 for 10 min, then washed. Nonspecific binding was blocked with 5% BSA for 30 min. The cells were incubated for 1 h with either an ETA or an ETB antibody (Alomone, Jerusalem, Israel; Murphy et al. 2011) diluted 1:100 in 5% BSA. Cells were then washed and incubated with a goat anti‐rabbit antibody labeled with Alexa Fluor 568 fluorescent dye (Alexa Fluor; Invitrogen, Carlsbad, CA), diluted 1:100 in 5% BSA for 1 h. After incubation with the secondary antibody, cells were again washed and then incubated for 1 h with a 1:50 dilution of an antibody against renin protein (sheep anti‐mouse FITC‐labeled; Innovative Research Inc., Novi, MI). Cells were again washed and the coverslips were mounted on slides with Fluoromount (Southern Biotech Associates Inc., Birmingham, AL). The preparations were examined by fluorescent microscopy (Nikon diaphot 300, Melville, NY). This protocol was repeated four times and each time images of at least 20 cells were taken.

#### Detection of ET receptors in freshly fixed mouse renal cortical slices

C57BL/6NJ male mice kidneys were preserved by in situ retrograde perfusion of the aorta with 150 mmol/L NaCl to flush the kidney, followed by a 15‐min perfusion with 4% paraformaldehyde in buffer containing 150 mmol/L NaCl and 10 mmol/L sodium phosphate (pH 7.4). Kidneys were stored in 4% formaldehyde until sectioning, at which point they were embedded in paraffin and cut into slices 6 *μ*m thick. Longitudinal and transverse sections of the cortex were obtained from different kidneys. To expose antigenic sites, fixed paraffin‐embedded slices were first deparaffinized with xylene and then hydrated gradually through 100% ethanol, then 95%, 70%, and finally distilled water. Each lasted for 5 min. Slides were air‐dried and incubated for 10 min with Tryton 0.01% at 37°C, after permeabilization slides were incubated for 30 min with 5% (bovine serum albumin (BSA) to block unspecific unions. Afterward, slides were incubated for 1 h at 37°C with a 1:50 dilution of an antibody against mouse ETA or ETB receptor protein (Alomone, Jerusalem, Israel), and then for 1 h at 37°C with a 1:100 dilution of secondary antibody (Alexa Fluor 568 goat antimouse IgG; Life Technologies, Grand Island, NY). ET receptor fluorescence was detected with an inverted microscope (model IX81; Olympus America, Center Valley, PA) with a digital camera (DP70) set at 568 nm excitation at a 40× magnification.

### JG cell TRPC1, 4, and 5 expression

#### RT‐PCR for TRPC 1, 4, and 5 in JG cells

RT‐PCR for the TRPC 1/4/5 isoforms was run using pipette‐selected isolated mouse JG cells. A “no‐template” control was used as a negative control. Isolated JG cells were resuspended in 1 mL of Tri reagent. One microgram of extracted total RNA was reverse transcribed at 37°C using Omniscript (Qiagen, Valencia, CA) and 2 *μ*L of mixture was taken for subsequent PCR. The following primers (NCBI accession number: NM_011643.2) were used to detect the TRPC1: Sense 5′cctgttattttagctgctcatc 3′ and Antisense 5′ taagttcaaacgctctcagaat 3′. The following primers (NCBI accession number: NM_016984.3) were used to detect the TRPC4: Sense 5′ tttccttactgcctttcagtta 3′ and Antisense 5′ cggtaattaagaatgatttcca 3′. The following primers (NCBI accession number: NM_009428.2) were used to detect the TRPC5: Sense 5′ aacaagttacaactcggctcta 3′ and Antisense 5′ aaaaggcaaatgataatgacag 3′.PCR was performed under the following conditions: 94°C for 2 min; 40 cycles of 95°C for 30 sec, 60°C for 40 sec, 72°C for 1 min, followed by a final extension at 72°C for 5 min. The reaction products were then held at 4°C. PCR products were run on a 2% agarose gel in 1× TBE. TRPC1, 4, and 5 give a PCR product at 238, 168, and 247 bp, respectively (Yu et al. 2011).

### Renin release protocols

#### To test if endothelin 1 (ET‐1) inhibits renin release (*n *=**5)

Isolated JG cells were treated with: (1) vehicle; (2) 1 nmol/L ET‐1; (3) 10 nmol/L ET‐1; or (4) 50 nmol/L ET‐1. Cells were incubated for 1 h, after which the media were collected for determination of renin release; then the cells were harvested for determination of total JG protein. The concentrations used are derived from previously published works describe renin inhibition (Moe et al. 1991; Kramer et al. 1994; Ackermann et al. 1995; Ritthaler et al. 1995).

#### To test if endothelin 2 (ET‐2) inhibits renin release (*n *=**6)

Isolated JG cells were treated with: (1) vehicle; (2) 1 nmol/L ET‐2; (3) 10 nmol/L ET‐2; or (4) 50 nmol/L ET‐2. Cells were incubated for 1 h, after which the media were collected for determination of renin release: then the cells were harvested for determination of total JG protein. The concentrations used are derived from previously published works describe renin inhibition (Moe et al. 1991; Kramer et al. 1994; Ackermann et al. 1995; Ritthaler et al. 1995).

#### To test renin response to ETA receptor blockade after stimulation with endothelin (*n *=**8)

We tested the effects of an ETA receptor blocker, BQ123 (EMD Chemicals, San Diego, CA) on renin release after stimulation of JG cells with 10 nmol/L ET‐1. Isolated JG cells were treated with: (1) vehicle; (2) 10 nmol/L ET‐1; (3) ET‐1 plus 0.5 *μ*mol/L BQ123 (Scholz et al. 1995); (4) ET‐1 plus 1 *μ*mol/L BQ123 (Bkaily et al. 2008); or (5) ET‐1 plus 5 *μ*mol/L BQ123 (Mumtaz et al. 2006). Cells were incubated for 1 h, after which the media were collected for determination of renin release; then the cells were harvested for determination of total JG protein.

#### To test renin response to ETB receptor blockade after stimulation with endothelin (*n *=**8)

We tested the effects of an ETB receptor blocker BQ788 (EMD Chemicals) on renin release after stimulation of JG cells with 10 nmol/L ET‐1. Isolated JG cells were treated with: (1) vehicle; (2) 10 nmol/L ET‐1; (3) ET‐1 plus 1 *μ*mol/L BQ788 (Mutafova‐Yambolieva and Westfall 1998); (4) ET‐1 plus 3 *μ*mol/L BQ123 (O'Donnell and Kay 1995); or (5) ET‐1plus 10 *μ*mol/L BQ788 (Adner et al. 1996). Cells were incubated for 1 h, after which the media were collected for determination of renin release; then the cells were harvested for determination of total JG protein.

#### To test renin response to ETA receptor blockade after ETB stimulation with an agonist (*n *=**8)

We tested the effects of the ETA receptor blocker BQ123 on renin release after stimulation of the ETB receptor with 10 nmol/L of a selective ETB agonist: Sarafotoxin (S6c; EMD Chemicals; Evans and Walker 2008a). Isolated JG cells were treated with: (1) vehicle; (2) 10 nmol/L S6c; (3) S6c plus 1 *μ*mol/L BQ123; or (4) S6c plus 5 *μ*mol/L BQ123. Cells were incubated for 1 h, after which the media were collected for determination of renin release; then the cells were harvested for determination of total JG protein.

#### To study IP3 inhibition with 2‐APB (*n *=**10)

To study if renin inhibition resulting from ET receptor activation is dependent upon the PLC/IP3 pathway, the IP3 inhibitor 2‐APB (EMD Chemicals; Rossi et al. 2006; Ortiz‐Capisano et al. 2013b) was used after activation of the ET receptor using either ET‐1 or ET‐2. Isolated JG cells were treated with: (1) vehicle; (2) 100 *μ*mol/L 2‐APB (Ortiz‐Capisano et al. 2013b); (3) 10 nmol/L ET‐1 or 10 nmol/L ET‐2; and (4) 10 nmol/L ET‐1 plus 2‐APB or 10 nmol/L ET‐2 plus 2‐APB. Cells were incubated for 1 h, after which the media were collected for determination of renin release; then the cells were harvested for determination of total JG protein.

#### To test stimulation of the ET receptor with ET‐1 or ET‐2 after TRPC channels blockade (*n *=**8)

To determine if the endothelin‐mediated entry of calcium was *via* the TRPC channel in JG cells, we used the TRPC blocker SKF‐96365 (Jan et al. 1999; Horinouchi et al. 2011; Ding et al. 2012; SKF; Enzo Life Biosciences, Farmingdale, NY) in the presence and absence of the ET receptors agonists ET‐1 or ET‐2. Isolated JG cells were treated with: (1) vehicle; (2) 50 *μ*mol/L SKF (Jan et al. 1999; Horinouchi et al. 2011); (3) 10 nmol/L ET‐1 or 10 nmol/L ET‐2; and (4) 10 nmol/L ET‐1 plus SKF or 10 nmol/L ET‐2 plus SKF. Cells were incubated for 1 h, after which the media were collected for determination of renin release; then the cells were harvested for determination of total JG protein.

### Assays

#### Renin release

After 1‐h JG cell incubation, the medium was drawn off, centrifuged, and the supernatant recovered for assay of renin concentration (AngI generation). Samples were incubated for 3 h with excess rat angiotensinogen as substrate, as previously described (Ortiz‐Capisano et al. 2009; Atchison et al. 2010; Mendez et al. 2011). Renin consumed <15% of exogenous substrate to ensure the enzymatic reaction remained in first‐order kinetics. Angiotensin I generation was assayed using a Gamma Coat RIA kit (DiaSorin, Stillwater, MN) as previously described (Ortiz‐Capisano et al. 2007a,b,c, 2009; Atchison et al. 2011; Mendez et al. 2011). Values for renin concentration (*μ*gAngI generated/mL sample/h of incubation) were corrected for JG cell total protein and are presented hereafter as *μ*gAngI/mL/h/mg prot.

#### Protein concentration

The protein concentration in JG cell lysates used to correct renin release/mg protein was determined using the Coomassie plus Protein Assay Reagent kit (Pierce Biotechnology Inc, Rockford, IL‎) according to the manufacturer's instructions, as previously described (Ortiz‐Capisano et al. 2007b, 2009).

### Statistical analysis

All data were derived and analyzed from paired control and experimental permutations in primary cultures from the same tissue pool obtained on a single day (*n *=**1) from the cortices of four mice. Changes in renin release (as determined from AngI production), compared to controls, were evaluated using either a paired *t*‐test (for a single comparison), or analysis of variance (ANOVA) for repeated measures with a Bonferroni post‐hoc test. We considered a corrected *P*‐value < 0.05 to be significant. In the Figures, for the sake of simplicity, all statistically significant changes are represented as *P *<**0.05. To normalize and simplify data presentation, figures show renin release from JG cells as a change expressed as a % of control. However, actual data are provided in the results narrative.

## Results

### RT‐PCR for ETA receptor

**Figure 1.**
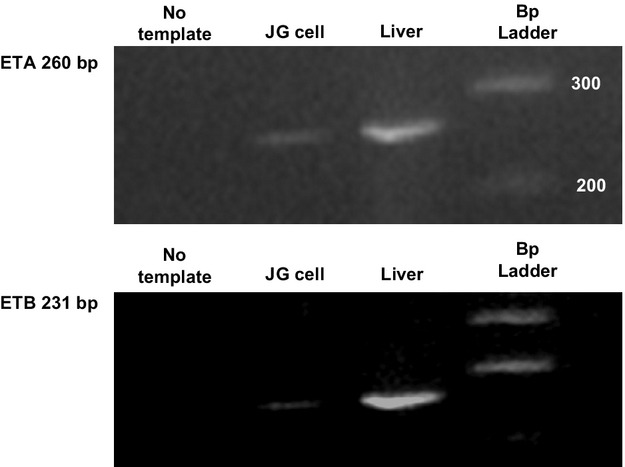
RT‐PCR for ETA (top) and ETB (bottom) identifying bands at 260 and 231 bp, respectively. Column 1 is the “no‐template” negative control. Column 2 is total mRNA obtained from isolated juxtaglomerular (JG) cells. Column 3 is total mRNA obtained from the positive control of liver. Column 4 is the calibration scale (100 bp ladder).

Figure 1(top) shows that RT‐PCR performed on 1 *μ*g of total RNA from JG cells gave a product at the expected size of 260 bp in both isolated JG cells and in the positive control of mouse liver. The “no‐template” negative control showed no amplification. These results suggest that there is expression of ETA in JG cells.

### RT‐PCR for ETB receptor

Figure 1(bottom) shows that RT‐PCR performed on 1 *μ*g of total RNA from JG cells gave a product at the expected size of 231 bp in both isolated JG cells and in the positive control of mouse liver. The “no‐template” negative control showed no amplification. These results suggest that there is expression of ETB in JG cells.

### Coimmunolabeling of ET receptors (A and B) and renin in JG cells

**Figure 2.**
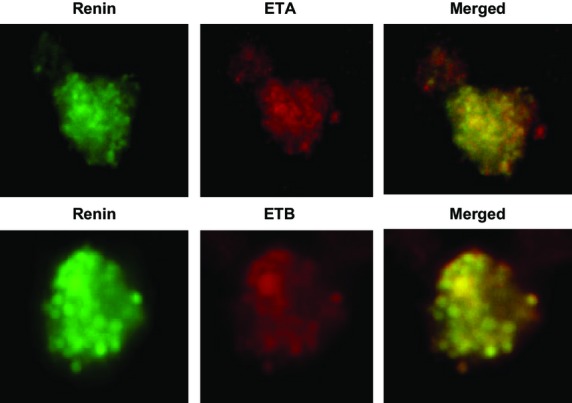
Immunofluorescence of a single juxtaglomerular (JG) cell using two antibodies; one specific for renin (in green) to confirm this is a JG cell, and another specific for the ETA (top) and ETB (bottom) receptors isoforms (in red). The third panel shows both renin and A1R in the same JG cell (merged image).

We used an ETA antibody and found positive labeling for the ETA isoform in JG cells grown on coverslips. Figure 2(top) shows a typical example in which the ETA receptor (shown in red) is localized in a single JG cell, as identified by positive labeling for renin (shown in green). The merged image (yellow) shows the same JG cell labeled with both antibodies.

Likewise, the same protocol was performed with an ETB receptor selective antibody and found positive labeling in the renin‐positive JG cells, (Fig. 2 bottom). ETB receptor (shown in red) is localized in a single JG cell, as identified by positive labeling for renin (shown in green). The merged image (yellow) shows the same JG cell labeled with both antibodies.

### Detection of ET receptors in freshly fixed mouse renal cortical slices

**Figure 3.**
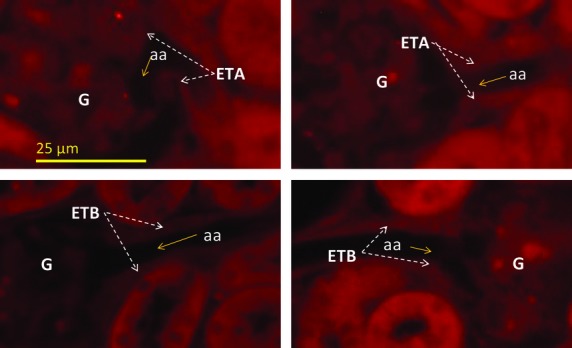
Immunoflourescence of mouse renal cortical sections fixed in situ showing (in red) ET receptors A (top) and ETB (bottom) in the afferent arteriole (aa) within the juxtaglomerular apparatus next to the glomerulus (G) using antibodies selective for each (see text).

Figure 3 represents freshly fixed renal cortical sections labeled for the ETA receptor (on the top) and cortical sections labeled for the ETB receptor (on the bottom). ET receptors (both ETA and ETB) are expressed throughout the proximal tubule as expected (Kohan et al. 2011), as well as the afferent arteriole (aa) next to the glomerulus (G), The immunolabeling demonstrates that both ETA and ETB receptors are expressed in the afferent arteriole (Barajas 1979) in mouse renal cortical slices fixed in vivo consistent with labeling in isolated primary cultures of JG cells.

### RT‐PCR for TRPC1/4/5

**Figure 4.**
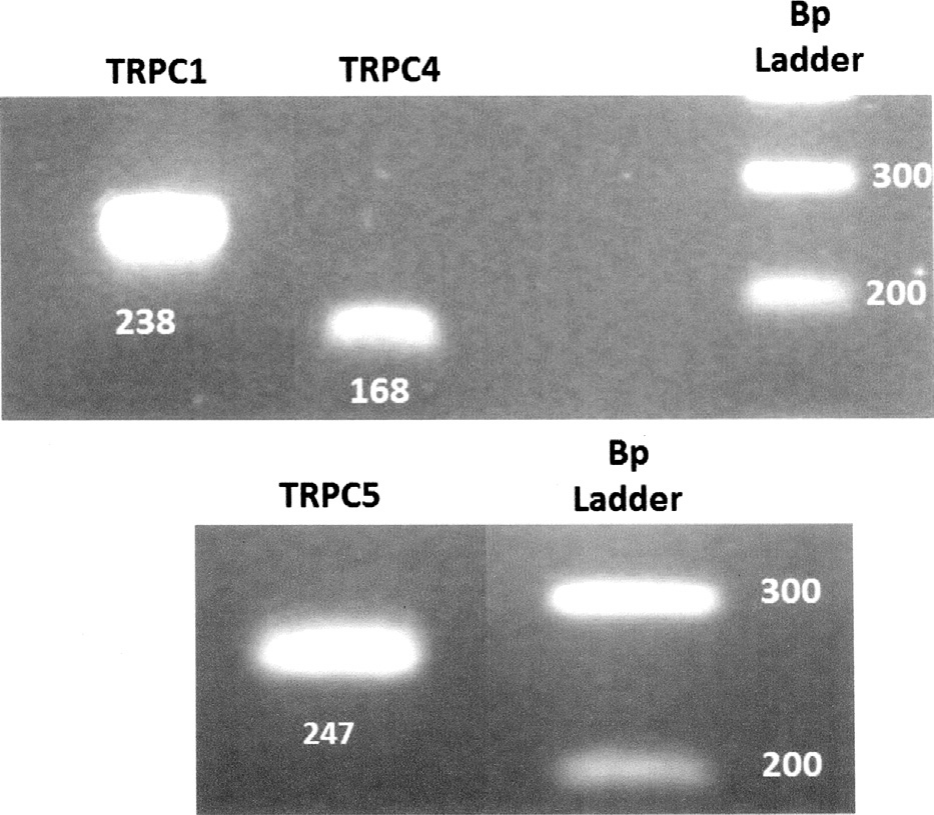
Two different gels; one for TRPC1 and TRPC4 (top) and another for TRPC5 (bottom) isoforms, run under the same conditions. RT‐PCR for TRPC 1, 4, and 5 identifying three bands at a 238, 168, and 247 bp. On the top columns 1 and 2 total mRNA obtained from isolated juxtaglomerular (JG) cells. Column 3 is the “no‐template” negative control. Column 4 is the calibration scale (100 bp ladder). On the bottom column 1 is total mRNA obtained from isolated JG cells. Column 2 is the “no‐template” negative control and column 3 is the calibration scale (100 bp ladder).

Figure 4 shows that the RT‐PCR performed on 1 *μ*g of total RNA gave positive products for TRPC1, 4, and 5 at the expected size 238, 168 (Fig. 4 top), and 247 bp (Fig. 4 bottom), respectively. The “no‐template” negative control showed no amplification. These results suggest that there is expression of TRPC1 and TRPC4 and TRPC5 in JG cells.

### Endothelin 1 and 2 inhibit renin release

Incubation of JG cells with 1 nmol/L ET‐1 decreased basal renin release by 30% from 0.30 ± 0.04 to 0.23 ± 0.03 *μ*gAngI/mL/h/mg prot. Incubation of JG cells with 10 nmol/L ET‐1 likewise decreased renin release to 0.20 ± 0.02 *μ*gAngI/mL/h/mg prot. (*P *<**0.05 vs. control). Using 50 nmol/L ET‐1 also decreased renin release to 0.23 ± 0.07 *μ*gAngI/mL/h/mg prot. All of the doses of ET‐1 inhibited renin release to a similar extent. Incubation of JG cells with 1 nmol/L ET‐2 decreased basal renin release by 23% from 0.35 ± 0.03 to 0.27 ± 0.01 *μ*gAngI/mL/h/mg prot. Incubation of JG cells with 10 nmol/L ET‐2 likewise decreased renin release to 0.24 ± 0.01 *μ*gAngI/mL/h/mg prot. (*P *<**0.05 vs. control). Using 50 nmol/L ET‐2 also decreased renin release to 0.25 ± 0.02 *μ*gAngI/mL/h/mg prot. (*P *<**0.05 vs. control). All of the doses of ET‐2 inhibited renin release to a similar extent.

### Renin response to ETA receptor blockade after stimulation with endothelin

**Figure 5.**
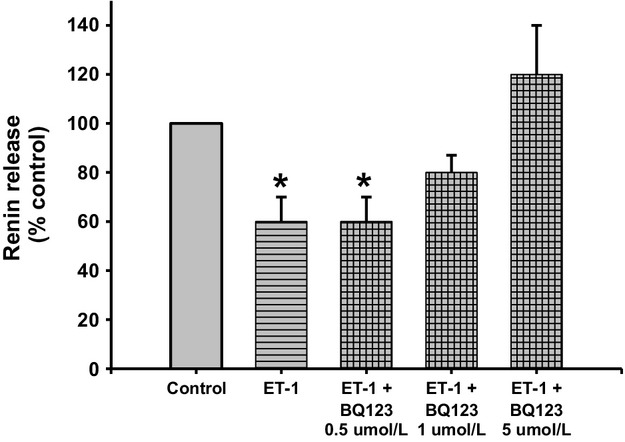
Renin release from juxtaglomerular (JG) cells under basal conditions (Control), after incubation with ET‐1 (10 nmol/L), or ET‐1 plus different concentrations of the ETA receptor blocker BQ123 (0, 5 *μ*mol/L, 1 *μ*mol/L and 5 *μ*mol/L). Incubation with ET‐1 reduced renin release. This response was inhibited by 5 *μ*mol/L of an ETA receptor blocker. *P <**0.05 versus control.

Renin release decreased by 40% when JG cells were incubated with 10 nmol/L ET‐1; from 0.30 ± 0.05 to 0.18 ± 0.03 *μ*gAngI/mL/h/mg prot (Fig. 5). (*P *<**0.05 vs. control). Renin release remained at reduced levels with 0.5 *μ*mol/L BQ123 to 0.18 ± 0.03 *μ*gAngI/mL/h/mg prot. (*P *<**0.05 vs. control). Increasing the concentration of BQ123 to 1 and 5 *μ*mol/L reversed the renin inhibition obtained with ET‐1 to 0.24 ± 0.20 and 0.36 ± 0.60 *μ*gAngI/mL/h/mg prot., respectively. Thus, ETA receptor stimulation with ET‐1 inhibits renin release.

### Renin response to ETB receptor blockade after stimulation with ET‐1

**Figure 6.**
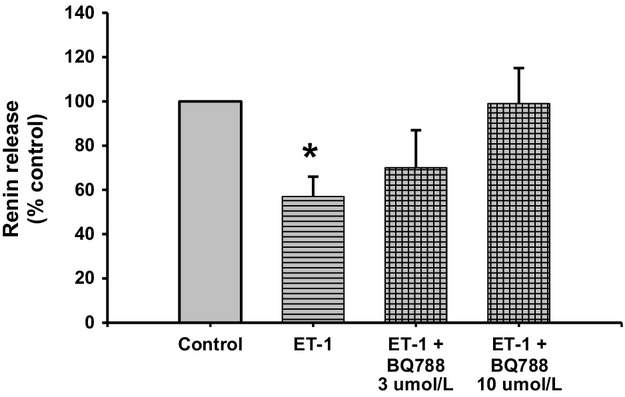
Renin release from juxtaglomerular (JG) cells under basal conditions (Control), after incubation with ET‐1, or ET‐1 plus different concentrations of the ETB receptor antagonist BQ788 (3 *μ*mol/L and 10 *μ*mol/L). Incubation with ET‐1 reduced renin release. This response was inhibited by 10 *μ*mol/L of an ETB receptor blocker. *P <**0.05 versus control.

Renin release decreased by 43% when JG cells were incubated with 10 nmol/L ET‐1; from 0.77 ± 0.12 to 0.44 ± 0.07 *μ*gAngI/mL/h/mg prot (Fig. 6; *P *<**0.05 vs. control). Incubation with the ETB receptor blocker BQ788 at 3 and 10 *μ*mol/L reversed the renin inhibition obtained with ET‐1 to 0.54 ± 0.13 *μ*gAngI/mL/h/mg prot. and 0.76 ± 0.12 *μ*gAngI/mL/h/mg prot., respectively. Thus, ETB receptor stimulation with ET‐1 inhibits renin release.

### Renin response to ETA receptor blockade after ETB receptor activation

**Figure 7.**
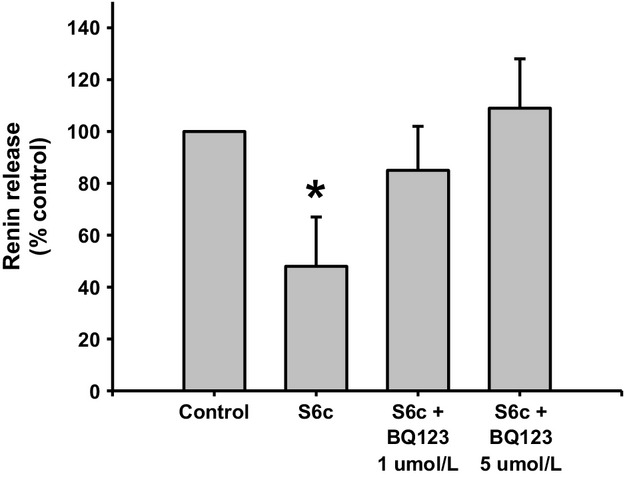
Renin release from juxtaglomerular (JG) cells under basal conditions (Control), after incubation with Sarafotoxin 10 nmol/L (S6c), or S6c plus two different concentrations of the ETA receptor antagonist BQ123 (1 *μ*mol/L and 5 *μ*mol/L). Incubation with S6c reduced renin release. This response was inhibited by 5 *μ*mol/L of an ETA receptor blocker. *P <**0.05 versus control.

Renin release decreased by 40% when JG cells were incubated with 10 nmol/L S6c; from 0.46 ± 0.09 to 0.22 ± 0.02 *μ*gAngI/mL/h/mg prot (Fig. 7; *P *<**0.03 vs. control). However, ETA receptor inhibition with both 1 and 5 *μ*mol/L BQ123 reversed the renin inhibition obtained with S6c to 0.39 ± 0.09 and 0.50 ± 0.13 *μ*gAngI/mL/h/mg prot., respectively. Thus, both ETA and ETB receptor stimulation with ET‐1 inhibits renin release.

### IP3 inhibition with 2‐APB

**Figure 8.**
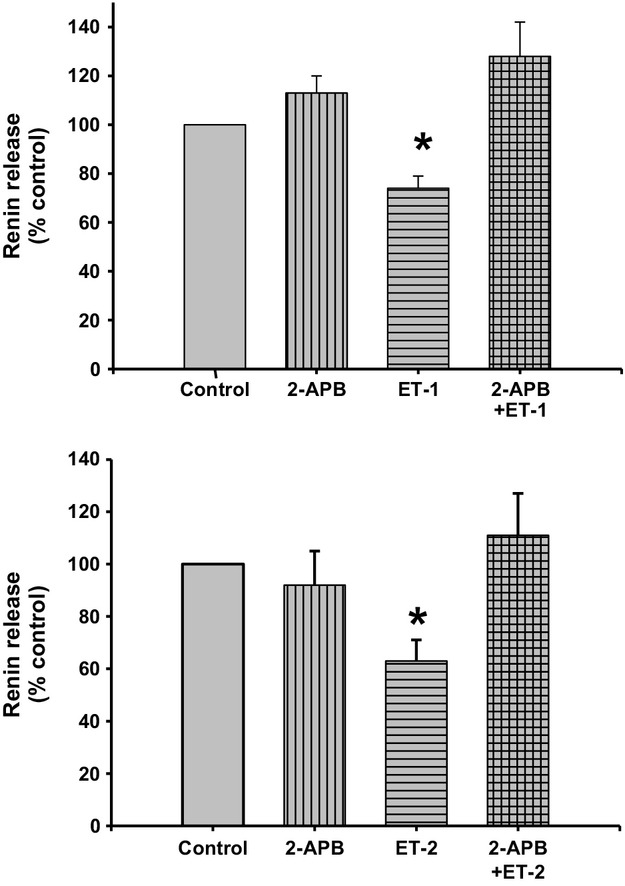
Renin release from juxtaglomerular (JG) cells under basal conditions (Control) or after incubation with ET‐1 (10 nmol/L) at the top, and ET‐2 (10 nmol/L) at the bottom. The IP3 inhibitor 2‐APB (100 *μ*mol/L) alone did not change basal renin release. ET‐1 and ET‐2 significantly decreased renin release, and incubation with both 2‐APB and either ET‐1 or ET‐2 returned renin release at values similar to control. *P <**0.05 versus control.

Incubation of JG cells with the IP3 blocker 2‐APB did not change basal renin release (0.37 ± 0.02 vs. 0.41 ± 0.03 *μ*gAngI/mL/h/mg prot; Fig. 8). Incubation of JG cells with 10 nmol/L ET‐1 decreased basal renin release by 30%, to 0.30 ± 0.03 *μ*gAngI/mL/h/mg prot (*P *<**0.05 vs. control). However, when JG cells were incubated with both ET‐1 plus 2‐APB, renin release returned to basal (0.48 ± 0.06 *μ*gAngI/mL/h/mg prot.), a result not different from JG cells treated with 2‐APB alone.

Likewise, repeating this experiments using ET‐2 produced a similar response (Fig. 8 bottom) where incubation of the JG cells with the IP3 blocker 2‐APB did not change basal renin release (0.36 ± 0.06 vs. 0.33 ± 0.05 *μ*gAngI/mL/h/mg prot.). Incubation of the JG cells with 10 nmol/L ET‐2 decreased basal renin release by 40%, to 0.23 ± 0.03 *μ*gAngI/mL/h/mg prot (*P *<**0.05 vs. control). However, when JG cells were incubated with both ET‐2 plus 2‐APB, renin release returned to basal, to 0.40 ± 0.04 *μ*gAngI/mL/h/mg prot. Thus, the inhibitory effect of endothelin in renin release involves a PLC/IP3 pathway activation.

### Stimulation of the ET receptor with ET‐1 or ET‐2 after TRPC channels blockade

**Figure 9.**
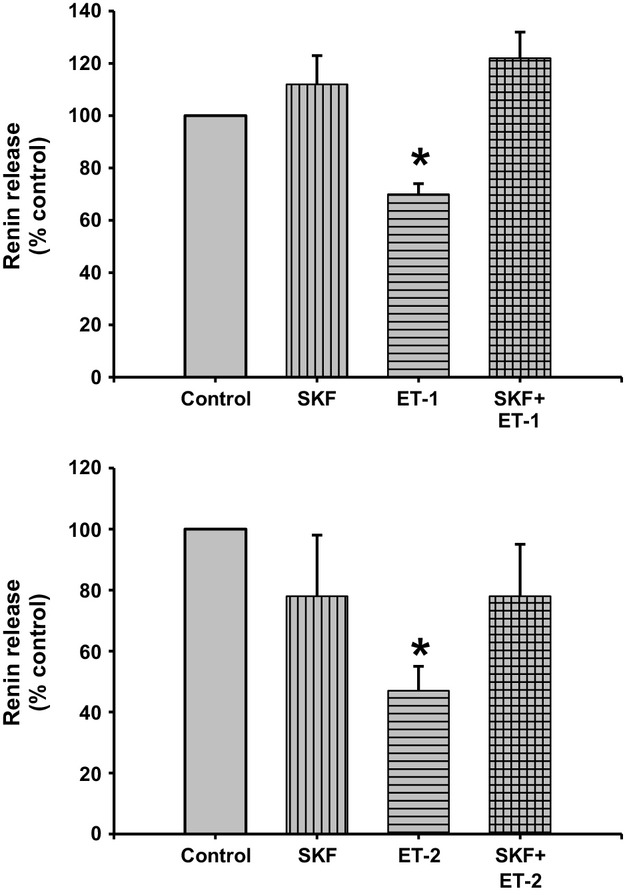
Renin release from juxtaglomerular (JG) cells under basal conditions (Control) or after incubation with ET‐1 (10 nmol/L) at the top and ET‐2 (10 nmol/L) at the bottom. The TRPC channel blocker SKF (50 *μ*mol/L) alone did not change basal renin release. ET‐1 and ET‐2 significantly decreased renin release, and incubation with both SKF and either ET‐1 or ET‐2 returned renin release at values similar to control, completely reversing endothelin inhibition of renin. *P <**0.05 versus control.

Incubation of the JG cells with TRPC channel blocker SKF did not change basal renin release (0.84 ± 0.07 vs. 0.92 ± 0.08 *μ*gAngI/mL/h/mg prot.; Fig. 9). Incubation of the JG cells with 10 nmol/L ET‐1 decreased basal renin release by 30%, to 0.60 ± 0.07 *μ*gAngI/mL/h/mg prot (*P *<**0.05 vs. control). However, when JG cells were incubated with both ET‐1 plus SKF, renin release returned to basal (0.99 ± 0.07 *μ*gAngI/mL/h/mg prot.); a result not different from JG cells treated with SKF alone.

Repeating this experiments using ET‐2 produced a very similar response. Incubation of the JG cells with the SKF did not change basal renin release (0.36 ± 0.06 vs. 0.28 ± 0.06 *μ*gAngI/mL/h/mg prot.). Incubation of the JG cells with 10 nmol/L ET‐2 decreased basal renin release by 47%, to 0.17 ± 0.03 *μ*gAngI/mL/h/mg prot (*P *<**0.05 vs. control). However, when JG cells were incubated with both ET‐2 plus SKF, renin release returned to values not different from basal (0.28 ± 0.04 *μ*gAngI/mL/h/mg prot.). Combined, these results show that blocking calcium entry *via* TRPC channels reversed the endothelin‐mediated inhibition of renin release by either ET‐1 or ET‐2.

## Discussion

I hypothesized that endothelin, *via* ETB receptor activation, inhibits renin release by triggering the TRPC store‐operated Ca channels to increase intracellular Ca. This would interact with the Ca‐inhibitable adenylyl cyclase 5 to inhibit cAMP‐stimulated renin release, as previously reported (Ortiz‐Capisano et al. 2007b,c). This work shows, for the first time, expression of both the ETA and ETB receptors in JG cells. Furthermore, that both receptors are involved in the ET‐mediated inhibition of renin release from JG cells. This inhibition is Ca‐mediated through either or both ET receptors, *via* a PLC/IP3 pathway that stimulates release of Ca from intracellular stores; activating the TRPC channels in a store‐operated manner.

The present work shows expression of both mRNA and protein for both ETA and ETB receptors in JG cells, as shown by RT‐PCR and immunofluorescence (see Figs. 1, 2). However, to ensure that their expression in vitro is not anomalous, it is also shown, by immunofluorescence that ET receptors localize in the area of the afferent arteriole (Fig. 3) where JG cells localize in mouse renal cortex in vivo (Kohan et al. 2011). Thus, the immunofluorescence results provide further evidence of the localization of the ET receptors on the JG cells.

The renal circulation, similar to most vascular beds, contains both ETA and ETB receptors. There also seems to be a heterogeneous distribution of ETA and ETB receptors over the length of the renal arterial system as revealed in isolated vascular preparations (Inscho et al. 2005). It is suggested that every cell type in the kidney may express ET receptors (Kohan et al. 2011), yet there have been no studies identifying either mRNA or protein expression of ETA or ETB in JG cells (Kohan et al. 2011). Functional data showed that an ETA antagonist (BQ123) had no effect in the ET1‐mediated inhibition of renin release from isolated JG cells (Ritthaler et al. 1995), but an ETB agonist (S6c) mimicked the inhibitory effect of ET on renin secretion from JG cells (Ritthaler et al. 1996). Thus, it was assumed that only the ETB receptor, but not ETA, was involved in endothelin‐mediated inhibition of renin release. The present work shows for the first time immunohistological evidence that both ETA and ETB receptors are expressed in the JG cells.

Since, it was previously reported (Ritthaler et al. 1995) that only the ETB receptor agonist but not the ETA receptor antagonist had an effect on renin release from JG cells, I originally proposed that the effect of endothelin activation of ETB receptors leads to TRPC channels opening, increasing intracellular Ca and inhibiting renin release. However, my results show that both ETA and ETB receptors can mediate inhibition of renin release to the same extent (see Figs. 5–7). In the original work of Ritthaler et al. (1995) the concentration of the selective ETA blocker was lower than 0.5 *μ*mol/L. The IC50 of BQ123 varies in different experimental models or cell types. For example, in cultured rat brain capillary endothelial cells its IC50 is 1.3 *μ*mol/L (Vigne et al. 1994), while in human adult mesangial cells it is 10 *μ*mol/L (Orth et al. 2000), and in porcine aortic smooth muscle cells it is 7.3 nmol/L (Ihara et al. 1992). Thus, in my experiments in isolated JG cells, repeating the concentration reported to have been used in JG cells (0.5 *μ*mol/L; Scholz et al. 1995) and 2 higher concentrations of 1 (Mumtaz et al. 2006; Bkaily et al. 2008) and 5 *μ*mol/L (Mutafova‐Yambolieva and Westfall 1998) were used in my design. While 0.5 *μ*mol/L BQ123 had no effect on ET‐mediated renin inhibition, both 1 and 5 *μ*mol/L blocked renin inhibition by ET (see Fig. 5). The selective ETB inhibitor BQ‐788 significantly blocked the renin inhibiting effects of ET‐1 (see Fig. 6). In order to confirm and differentiate which ET receptor is more prevalent, JG cells were incubated with the ETB receptor agonist (S6c; Ritthaler et al. 1995) and ETA blocked with BQ‐123. Results showed that both ETA and ETB receptors mediate the effects in endothelin inhibition of renin release (see Fig. 7).

It was unexpected to find that both receptors blockers had the same potency in reversing the endothelin‐mediated inhibition of renin release. Does this mean that they act *via* the same pathway? It is very likely that this is the case. It has been reported that ETA and ETB receptors can form heterodimers (Rapoport and Zuccarello 2011; Yatawara et al. 2013), and further that they are trafficked to the plasma membrane as monomers and through constitutive dimer formation they form the heterodimers in the plasma membrane. Upon activation with 10 nmol/L ET‐1 they increase intracellular Ca (Evans and Walker 2008a,b). The present work shows for the first time that ETA and ETB receptors can both mediate inhibition of renin release to the same extent.

This study tested the possibility that ET receptor acting as Gq‐protein‐coupled receptor induced PLC, leading to the generation of IP3, releasing calcium from intracellular stores (Volpe et al. 1990). When JG cells were incubated with the IP3 blocker, the renin‐inhibitory effect of ET was blocked. Thus, it is likely that this is the mechanism through which ET inhibits renin release. These results were expected, since ET receptors couple to members of the Gi, Gq, Gs, and G*α*12/13 G‐protein families to regulate a variety of signaling cascades (Kohan et al. 2011). Gq_/_subtype protein activates phospholipase C (PLC). This results in increased membrane phosphatidyl inositol biphosphate (PIP_2_)‐turnover, and production of inositol‐1,4,5‐triphosphate (IP3) and diacylglycerol (DAG). Activation of IP3 receptors on the endoplasmic reticulum leads to Ca release from intracellular stores (Murphy et al. 2011). The increase in intracellular Ca suppresses the calcium‐inhibitable adenylyl cyclase isoform 5 (Ortiz‐Capisano et al. 2007b), possibly also isoform 6 (Grunberger et al. 2006; if present), and by activating the calcium‐stimulated phosphodiesterase 1C (Ortiz‐Capisano et al. 2009). Thus, increased intracellular Ca leads to suppression of synthesis and enhanced degradation of cAMP, the dominant cyclic nucleotide second messenger regulating renin secretion (Churchill 1985; Kim et al. 2012). For the first time, these studies show that the ET‐mediated inhibition of renin release involves activation of a PLC/IP3‐mediated pathway.

Endothelin receptor activation of the JG cell results in TRPC channel‐mediated calcium entry and the resulting calcium‐mediated inhibition of renin release. The canonical transient receptor potential (TRPC) channels can be activated by the activation of PLC by a G‐protein‐coupled (G) pathway, leading to the production of IP3, which activates the IP3 receptor (IP3R) causing release of Ca from the endoplasmic reticulum. This, in turn, activates TRPC channels via a store‐operated pathway (Putney 2005; Putney and Tomita 2011). Recently published data from this laboratory described TRPC 3 and 6 expression in isolated JG cells and their involvement in Ca‐mediated inhibition of renin release in an adenosine‐dependent system (Ortiz‐Capisano et al. 2013a). In this study, RT‐PCR showed expression of TRPC isoforms 1, 4, and 5.

To test the involvement of TRPC in the endothelin‐mediated inhibition of renin release, a nonselective TRPC inhibitor, SKF‐96365 (Ortiz‐Capisano et al. 2013a), was used with incubation of JG cells with both ET‐1 and ET‐2 (see Fig. 9). TRPC channel inhibition completely reversed ET‐1 and ET‐2‐mediated decreases in renin release. There are several studies showing that endothelin, *via* ETA and ETB receptor activation, activates TRPC channels (Saleh et al. 2009; Horinouchi et al. 2011; Adebiyi et al. 2012); Large et al. (Saleh et al. 2009) showed that stimulation of ETA and ETB receptors activate TRPC1 channels through two distinct phospholipid pathways in rabbit coronary artery myocytes. Thus, one could conclude that both ETA and ETB receptors, both expressed in JG cells, are involved in the ET‐mediated inhibition of renin release *via* TRPC channels activation. Understanding how endothelin inhibits renin by increasing intracellular Ca was the aim of the present work. The possibility that Ca‐mediated renin inhibition by ETs is linked to the activation of TRPC channels is a novel concept helping explain the specific intercellular cascade through which ETs act.

In summary, I have showed that JG cells express both the ETA and ETB receptors; that their activation leads to a calcium‐mediated inhibition of renin release, and that ET receptor activation involves a cascade via PLC/IP3 Ca release from intracellular stores, opening store‐operated TRPC calcium channels.

## Acknowledgment

Special thanks to T.‐D. Liao at Henry Ford Hospital for his help with fluorescent microscopy of freshly fixed renal cortex (Fig. 3).

## Conflict of Interest

None declared.
